# Efficient Delivery of Transducing Polymer Nanoparticles for Gene-Mediated Induction of Osteogenesis for Bone Regeneration

**DOI:** 10.3389/fbioe.2020.00849

**Published:** 2020-08-05

**Authors:** Aveen R. Jalal, James E. Dixon

**Affiliations:** Regenerative Medicine and Cellular Therapies Division, The University of Nottingham Biodiscovery Institute (BDI), School of Pharmacy, University of Nottingham, Nottingham, United Kingdom

**Keywords:** GET, CPP, PLGA NPs, gene delivery, bone regeneration, MSCs, pDNA

## Abstract

Developing non-viral gene therapy vectors that both protect and functionally deliver nucleic acid cargoes will be vital if gene augmentation and editing strategies are to be effectively combined with advanced regenerative medicine approaches. Currently such methodologies utilize high concentrations of recombinant growth factors, which result in toxicity and off-target effects. Herein we demonstrate the use of modified cell penetrating peptides (CPPs), termed Glycosaminoglycan (GAG)-binding Enhanced Transduction (GET) peptides with plasmid DNA (pDNA) encapsulated poly (lactic-co-glycolic acid) PLGA nanoparticles (pDNA-encapsulated PLGA NPs). In order to encapsulate the pDNA, it was first condensed with a cationic low molecular weight Poly L-Lysine (PLL) into 30–60 nm NPs followed by encapsulation in PLGA NPs by double emulsion; yielding encapsulation efficiencies (EE) of ∼30%. PLGA NPs complexed with GET peptides show enhanced intracellular delivery (up to sevenfold) and transfection efficiencies (up to five orders of magnitude). Moreover, the pDNA cargo has enhanced protection from nucleases (such as DNase I) promoting their translatability. As an example, we show these NPs efficiently deliver pBMP2 which can promote osteogenic differentiation *in vitro*. Gene delivery to human Mesenchymal Stromal Cells (hMSCs) inducing their osteogenic programming was confirmed by Alizarin red calcium staining and bone lineage specific gene expression (Q RT-PCR). By combining simplistic and FDA-approved PLGA polymer nanotechnology with the GET delivery system, therapeutic non-viral vectors could have significant impact in future cellular therapy and regenerative medicine applications.

## Introduction

Mesenchymal stromal cells (MSCs) are the most employed precursor cell lineage for advanced bone tissue regenerative strategies. Studies which use biomaterials along with exogenous MSCs or those that rely on their endogenous populations have shown significantly enhanced bone formation in critical-size defects when exogenous growth factors (GFs) are applied (Baylink et al., 1993; Kolambkar et al., 2011; Raftery et al., 2019). Numerous GFs have been identified as bone inducer molecules, amongst them bone morphogenetic proteins (BMPs) are the most widely employed GF family with BMP2 and BMP7 being the most prolifically applied FDA approved GFs used clinically for orthopedics (Urist, 1965; Lee, 1997; Groeneveld and Burger, 2000). Bone tissue regeneration through the delivery of recombinant human (rh)BMP2 such as commercially available INFUSE^®^ Bone Graft is a well-established method for spinal fusion and off-label applications, however protein instability (with degradation soon after administration) (Shields et al., 2006; James et al., 2016) means there is a need to apply supra-physiological concentrations to maintain efficacy within the therapeutic window, leading to safety concerns. These high levels of BMP2 are associated with serious side effects such as ectopic bone formation, osteolysis, aberrant immune responses and neurotoxicity (Lewandrowski et al., 2007; Tannoury and An, 2014). Therefore, there is a need to develop novel strategies to apply the activity of BMP2 more safely, physiologically and cost-effectively to promote bone healing in challenging and critical-sized bone traumas.

Triggering MSCs to produce BMP2 at more physiological levels, either from their own genome or via delivered exogenous transgenes is an alternative approach which does not rely on recombinant proteins. This can be achieved through upregulation of the bone differentiation gene regulatory network by transcription factors (Thiagarajan et al., 2017) or by direct gene delivery to express new BMP2 copies. In this case, the lower level of produced protein from transfected cells (picograms-nanograms) (Raftery et al., 2019) is more physiologically relevant than current approaches such as application of rhBMP2 (using milligrams per application). These lower levels result in minimal or no off-target effects when applied *in vivo* (Raftery et al., 2019). Nevertheless, the amount of published work in this field, using non-viral gene delivery for orthopedic regenerative medicine is limited mainly because MSCs are difficult to transfect cells and gene delivery is not easily translated especially *in vivo* (Lutz et al., 2008; Lee et al., 2010; Wegman et al., 2011; Park et al., 2019). Viral gene delivery methods are efficient but are expensive, are limited in insert size, can be insertionally mutagenic and sometimes are immunogenic which restricts their use (Zheng et al., 2000). Most non-viral gene delivery systems however are not effective in delivering the levels and efficiency of transfection that are required for therapeutic effect (Yin et al., 2014). Viral systems have evolved to effectively navigate the internal cellular barriers to gene delivery, however endosomal escape of the cargo with non-viral systems is a further inhibitory facet preventing effective use (Grigsby and Leong, 2010; Fihurka et al., 2018). Moreover, simplistic non-viral formulations using cationic molecules with nucleic acids (such as plasmid DNA; pDNA) which electrostatically form delivery nanoparticles (NPs) do not protect nucleic acid cargoes from enzymatic degradation by nucleases (Yang, 2011; Mathur and Medintz, 2019). It is widely known that polymers used to produce ‘hard’ NPs such as poly (lactic-co-glycolic acid) (PLGA) can protect encapsulated nucleic acids from nuclease-mediated degradation (such as by DNases) (Hirosue et al., 2001; Abbas et al., 2008). Moreover, the use of PLGA polymers can improve other aspects of non-viral gene delivery such as promoting physical stability and prolonged storage for industrial scalability and pharmaceutical level production (Kapoor et al., 2015; Sharma et al., 2016).

Glycosaminoglycan-binding Enhanced Transduction (GET) peptides (Dixon et al., 2016; Eltaher et al., 2016; Abu-Awwad et al., 2017; Thiagarajan et al., 2017; Osman et al., 2018; Markides et al., 2019; Raftery et al., 2019; Spiliotopoulos et al., 2019) are delivery peptides that enhance transduction and endosomal escape of cargoes in cells. They contain Heparan Sulfate (HS) binding motifs that enhance binding to cell surface glycosaminoglycans (GAGs) which concentrate the cargo on cell membranes, combined with cell penetrating peptide (CPP) and endosomal escaping peptide elements to mediate uptake and escape, respectively. We have previously published the enhanced delivery characteristics and versatile use of GET peptides, with sequences termed PR (P218R), PLR (P21LK158R), and FLR (FGF2BLK158R). These sequences can be used as synthesized L-amino acid peptides or recombinant protein fusions to deliver red fluorescent protein (mRFP), pDNA, mRNA, siRNA (Dixon et al., 2016), and magnetic NPs (Markides et al., 2019). Importantly we have shown GET’s utility in regenerative medicine by delivering transcription factors RUNX2 and MYOD for osteogenesis and zonal myogenesis in three−dimensional gradients (Eltaher et al., 2016; Thiagarajan et al., 2017), respectively. Moreover, GET peptides have been used to enhance the delivery and transfection of nucleic acids for lung gene therapy and bone regeneration *in vivo* (Osman et al., 2018; Raftery et al., 2019). The later delivering GF genes to enhance the repair of a critical size calvarial bone defect in rats (Raftery et al., 2019).

Here in, we aimed to encapsulate pDNA in a PLGA NP core to promote enzymatic protection and enhanced physical stability, and then deliver these to cells by complexation with GET peptides on the surface for superior delivery efficiency. Initially, we applied this system to deliver and transfect secreted reporter *gaussia* luciferase (*gluc*)-expressing pDNA as a proof of concept, we then tested applicability in osteogenesis and using MSCs *in vitro* by encapsulation and delivery of BMP2 expressing pDNA (pBMP2). We demonstrate that the combination of GET peptide transfection agents, with the pharmaceutical properties of PLGA NPs can be highly effective for gene therapy, can help protect nucleic acid cargoes, and is a significant step in developing future gene augmentation and editing strategies for use in regenerative medicine.

## Materials and Methods

### Plasmid Preparation

For luciferase assays, reporter plasmid (pDNA) expressing *gaussia* luciferase (*gluc*) was acquired from New England Biolabs (pGluc). For osteogenic differentiation assays, human *BMP2* expressing pDNA (pBMP2) was a kind gift from Royal College of Surgeons in Ireland (RCSI) and that used in Raftery et al. (2019). Both plasmids are driven by an enhanced cytomegalovirus (CMV) promoter. The plasmids were transformed in DH5α competent *E. coli* cells and purified by Maxi-prep kits (Qiagen, United Kingdom) as previously (Raftery et al., 2019).

### Condensation of pDNA With PLL

The pGluc vector (5764 bp) or pBMP2 vector (3486 bp) (100 μg) was complexed with 500 μg of Poly L-Lysine hydrobromide (PLL) (1000–5000 Da, Sigma, United Kingdom) by sequentially adding 10 μg of pDNA dropwise to 50 μg of PLL using a P200 pipette tip under vortexing. The working concentration was 40 and 200 μg/ml in nuclease-free water for pDNA and PLL, respectively. The mixture of pDNA-PLL NPs in a volume of 5 ml was then concentrated to 250 μl using Amicon^®^ Ultra Centrifugal Filter units. This volume of the mixture of pDNA-PLL NPs was encapsulated in the PLGA NPs by double emulsion as below. The hydrodynamic size and surface charge of pDNA-PLL NPs were assessed by Malvern Zetasizer Nano ZS. NP morphology was determined by Transmission Electron Microscopy (FEI Tecnai BioTwin-12 TEM) as previously (Osman et al., 2018).

### Preparation of pGluc and pBMP2 Encapsulated PLGA NPs

Double emulsion (W1/O/W2) was applied to encapsulate pDNA-PLL NPs (W1) into PLGA NPs. 10 mg of PLGA (with terminal carboxyl group, PLGA, 50:50, Mw 52000Da, Rosemer Evonic) dissolved in Dichloromethane (DCM) at concentration of 1.3% (w/v) was used as the oil phase (O). An aqueous solution of 0.5% (w/v) Bovine Serum Albumin (BSA) was applied as the inner phase surfactant. The aqueous phase (pDNA-PLL NPs + BSA solution) was sonicated in the oil phase for 20 s at 70% amplitude and 70% time on cycle on ice. This emulsion was immediately poured into a 3% PVA (w/v) (86–89% hydrolyzed, medium molecular weight, Alfa Aesar) aqueous solution (W2) and sonicated again at the same rate. After evaporation of DCM, the pDNA-encapsulated PLGA NPs were collected by centrifugation at 20,000 *g* for 20 min at 4°C and washed twice with deionized water to remove excess PVA and unbound pDNA. The NPs were re-suspended in 500 μl of 1.6% (w/v) Trehalose solution in a ratio of 1:1.5 by weight, respectively, then freeze dried and stored at −20°C until further use. To prepare fluorescently labeled PLGA NPs, hydrophilic and hydrophobic fluorescent dyes: Atto590 or Nile Red (1 mg) was encapsulated instead of pDNA-PLL NPs.

### Measurement of EE%

To measure the encapsulation efficiency percentage (EE%), 5.5 mg of pDNA encapsulated PLGA NPs were dissolved in 1 ml DCM. 1 ml of 1x TE buffer (Tris-EDTA, Thermo Fisher Scientific) aqueous solution was added to the dissolved PLGA NPs to extract the encapsulated pDNA. The immiscible mixture of DCM/aqueous layer was separated by brief centrifugation. The aqueous layer was then transferred and the EE% was measured by PicoGreen in which the amount of pDNA detected form the solubilized PLGA NPs was subtracted from the total amount of pDNA used. A pDNA-PLL NP solution was used to construct a standard curve (Gebrekidan et al., 2000; Ribeiro et al., 2005). The amount of pDNA-PLL was calculated using Quant-iT PicoGreen dsDNA Assay Kit (Life Technologies).

### pDNA Integrity and DNase Protection Assay

To determine the effect of sonication on supercoiling and integrity of pDNA, sonicated and non-sonicated pDNA and pDNA-PLL NPs (as controls) and pDNA-PLL extracted from PLGA NPs was quantified and transformed into competent DH5α *E. coli*, with the number of bacterial colonies were compared as a quality measure. Moreover, to confirm the pDNA protection ability of PLGA NPs, 0.5 μg of naked or encapsulated pDNA was incubated with increasing concentrations of DNase I at 37°C for 15 min. DNase I was prepared by diluting DNase I stock solution of 2.72 U/μl to 0.025 U/μl, 0.0025 U/μl and 0.00025 U/μl in DNase buffer according to the manufacturer’s instruction (RNase-Free DNase kit, Qiagen). The buffer was 2.5 mM Magnesium chloride and 1 mM Calcium chloride in PBS. The digestion reaction was stopped by adding EDTA at 0.5M final concentration. Naked pDNA and pDNA-encapsulated PLGA NPs were run on 1% (w/v) agarose gel in 1x TAE buffer with Ethidium bromide (EtBr) and imaged on a Luminescence Image Analyser (LAS-4000, Fuji).

### GET Complexation With PLGA NPs

Blank, fluorescent dye- (Atto590 or Nile Red) or pDNA-encapsulated freeze-dried PLGA NPs were resuspended in PBS and complexed with GET peptide at different concentrations. The mixture was left to electrostatically interact for 15 min at room temperature. The complexation was confirmed by a shift in the surface charge of PLGA NPs measured by Zetasizer Nano ZS as previously described (Osman et al., 2018). This procedure was applied to produce PLGA-GET complexes for enhanced delivery and transfection, as well as differentiation studies.

To assess enhanced delivery with PR, PLR, or FLR peptides, fluorescent dye-encapsulated PLGA NPs were used. This mixture of PLGA-GET was added to pre-seeded, washed NIH3T3 or IHMSCs (Immortalized human Mesenchymal Stromal Cells) (Thiagarajan et al., 2017) in a total volume of 250 μl of growth media (GM; 10% fetal calf serum; FCS) or serum free media (SFM).

### Assessment of Enhanced Intracellular Delivery With PLGA-GET Complex

To demonstrate the enhanced delivery of PLGA NPs intracellularly, hydrophilic dye Atto590 and hydrophobic dye Nile Red encapsulated PLGA-GET NPs were delivered to NIH3T3 or IHMSCs cells. The complex was incubated overnight with the cells in either GM or SFM. The following day, transduced cells were washed twice with PBS and imaged by fluorescent microscopy (Leica DM IRB) using a green laser before being processed for flow cytometry. For flow cytometry, cells were trypsinized with trypsin/EDTA [0.25% (w/v) trypsin/2 mM EDTA] and fixed with 4% (w/v) paraformaldehyde (PFA). PLGA-GET cellular internalization was quantified using a Beckman Astrios Cell Sorter and 590 nm laser (20,000 cells, gated on untreated cells by forward/side scatter). Mean fluorescence intensity was used for statistical analysis.

### Assessment of Enhanced Transfection With PLGA-GET Complex

To assess enhanced transfection, pGluc (*gluc* encoded pDNA)-encapsulated PLGA NPs were complexed with variants of GET peptides; PR, PLR or FLR. These NPs were incubated with NIH3T3 cells overnight. Luciferase signal was measured the next day by luminometer and compared with controls (as previously described) (Raftery et al., 2019). Dose of GET peptide per mg of PLGA NPs was optimized based on transduction efficiency, transfection levels and cell viability studies.

### Cell Culture

Cells were cultured as described previously and incubated at 37°C and 5% CO_2_ (Skarn et al., 2014; Thiagarajan et al., 2017; Osman et al., 2018). Both NIH3T3 and IHMSCs (TERT and E6/7 immortalized in-house) were used for transduction, transfection and viability assessments of PLGA-GET NPs. IHMSCs were specifically used to deliver pBMP2-PLL encapsulated PLGA NPs and pBMP2-FLR for osteogenic differentiation assays as comparators. The IHMSCs were maintained in expansion media; Dulbecco’s modified Eagle medium (DMEM) supplemented with 20% (v/v) FCS, 2 mM L-glutamine, 100 units/ml penicillin, and 100 mg/ml streptomycin and 1% (v/v) Antibiotic/Antimycotic. The cells were passaged at 80% confluency and used at passages below 20. For intracellular delivery, transfection and differentiation studies, NIH3T3 and IHMSCs were seeded at a density of 100K and 35K per well in 24-well plate culture dishes, respectively. The cells were then incubated overnight to allow attachment. Before delivery of NPs, media was replaced, and the cells were washed twice with PBS. Two hundred microliter of GM or SFM media was added then completed with 50 μl transfection media. The transfection media was left for 3 days and media then replaced with 500 μl of the relevant media.

### Metabolic Activity Assays

Metabolic activity of IHMSCs were detected after transfection with pDNA encapsulated PLGA-GET NPs using PrestoBlue assay. Two hundred microliters of 10% (v/v) of the reagent in HBSS (Hanks’ Balanced Salt solution, Thermo Fisher Scientific) was added to 24-well plate. Prior to the addition of the reagent, the old media was removed, and the cells were washed with PBS trice. The reagent was left for 20–25 min and the fluorescence was measured on a plate reader (Infinite PRO, TECAN) at 560/590 nm.

### Osteogenic Differentiation Assays

The osteogenic assays were first optimized and adapted for FLR transfection. pBMP2-FLR NPs were generated by mixing pBMP2 with FLR at a charge ratio (±) of 6 and a peptide final concentration of 2 μM in serum reduced media (OptiMEM, Life technologies) as described previously (Raftery et al., 2019). IHMSCs were seeded, incubated overnight and transfected with 0.5 μg pBMP2-FLR NPs. DMEM/F12 Ham 1:1 (Life Technologies, United Kingdom) supplemented with 10% (v/v) FCS, 2 mM L-glutamine, 100 units/ml penicillin, and 100 mg/ml streptomycin, 50 μg/ml L-ascorbic acid 2-phosphate sesquimagnesium salt hydrate (Sigma, United Kingdom) and 10 mM *b*-glycerophosphate disodium salt pentahydrate (Acros Organics, United Kingdom) was used as basal media (BM). Osteopermissive (OP) and Osteoinductive (OI) media contained low (10 nM) and high (100 nM) Dexamethasone (Dex) in BM, respectively (Thiagarajan et al., 2017). These media combinations were used to test the effectiveness for pBMP2 delivery to induce osteogenic differentiation. For osteogenic differentiation with pBMP2-encapsulated PLGA NPs, 200 μg of PLGA NPs equivalent to 1 μg of encapsulated pBMP2 content was complexed with FLR at a final concentration of 4 μM FLR. In both cases of pBMP2-FLR and pBMP2-encapsulated PLGA-FLR NPs, the cells were delivered with a single transfection in OP media.

### Alizarin Red Staining

At week three, cells were analyzed microscopically for extracellular matrix and bone nodule formation. At week four, the osteogenic cultures were stained for calcium deposition using 2% (w/v) Alizarin Red solution (Alfa Aesar). To do this, cultures were washed three times with PBS and fixed with 4% (w/v) PFA followed by the removal of PFA and washing with deionized water three times. The cultures were stained for 5 min using 200 μl Alizarin Red solution. The excess Alizarin Red solution was removed and the cultures were washed with deionized water. This was followed by incubation in deionized water for 2 h to remove the background staining. For calcium quantification, the stained cultures were washed three times with deionized water and Alizarin Red was extracted by the addition of 200 μl 10% (v/v) acetic acid (Sigma) for 30 min while shaking. The eluted stain was transferred to microfuge tubes and heated at 85°C for 10 min, cooled and neutralized with 10% (w/v) ammonium hydroxide (Sigma). The absorbance was read at 405nm on a plate reader (Infinite PRO, TECAN) (Gregory et al., 2004). Fold increase in molar concentration was compared to controls maintained in expansion media, basal media, OP or OI media (Gregory et al., 2004).

### Gene Expression Analysis

Quantitative reverse transcription polymerase chain reaction (Q RT-PCR) was used to detect the level of osteogenic gene upregulation. Total RNA was extracted from differentiated IHMSCs using RNeasy kits (Qiagen). On column DNase I treatment was applied using RNase-free DNase kits (Qiagen). 0.4 μg total RNA was reverse transcribed in 20 μl reaction using SuperScript III Reverse Transcriptase (Life technologies) according to manufacturer’s protocol. Q RT-PCR reactions were performed using Taqman assays (Applied Biosystems, Life technologies). Osteogenic genes (*ALP–Hs01029144_m1; RUNX2–Hs00231692_m; BGLAP–Hs01587814_g1; SPP1–Hs00959010_m1*) were detected against *B-actin* (*ACTB-Hs99999903-m1)* reference gene. Relative expression level was calculated using ΔΔC_*T*_ method. Five biological replicates with three technical replicates were performed for each treatment.

### Statistical Analysis

Statistical analysis and graphs were generated using GraphPad Prism software package. Unpaired *t*-test and One-way Anova were used to determine significant variances between two groups or more respectively. Two-way Anova was used for grouped data. One-way and two-way Anova was followed by Tukey test to determine significance between each mean in multiple comparison. The data represented as mean ± SD. Variances between means were considered statistically significant with *p*-values: 0.0332 (^∗^), 0.0021 (^∗∗^), 0.0002 (^∗∗∗^), and <0.0001 (^****^). Experimental numbers were a minimum of five biological replicates, with three technical replicates for all data except NP characterization which were minimum of three biological replicates and three technical replicates.

## Results

### Characterization of pDNA-PLL NPs

**FIGURE 1:**
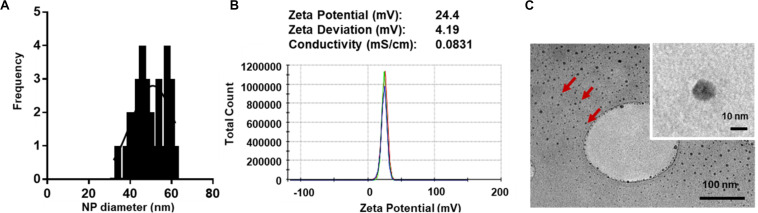
Characteristics of pDNA-PLL NPs. **(A)** NP size measured with Zetasizer Nano shows relatively small and homogenous distribution of NPs. **(B)** An image of a Zeta potential report indicates positive surface charge of NPs. The NPs were prepared and measured in nuclease free water; the same condition used for encapsulation in the double emulsion process. **(C)** TEM images demonstrate size and morphology of the same NPs. Red arrows highlight individual NPs.

For macromolecular hydrophilic and negatively charged plasmid DNA (∼7 megaDa) to be encapsulated into hydrophobic and relatively small 350 nm PLGA NPs, it is necessary for the pDNA to be condensed into a smaller size in proportion to the final PLGA NPs. We also aimed for pDNA to be converted to a more water insoluble and positively charged form to allow for more effective encapsulation. To do so, we used low molecular weight PLL (1–5 kDa) that is efficient in condensing large pDNA molecules. It is accepted that low molecular weight polycations can condense pDNA at charge ratio (±) equal or higher than 1. In this study, we have condensed pGluc and pBMP2 at weight ratio of 5 and charge ratio of 12 (PLL/pDNA) to produce small 30–60 nm (Figures 1A,C) and positively charged pDNA-PLL NPs (Figure 1B). These NPs are relatively uniformly distributed with an average Poly Dispersity Index (PDI) of 0.1 ± 0.03 (mean ± SD) (Wolfert and Seymour, 1996). Moreover, these NPs are spherical in morphology as showed on TEM images (Figure 1C).

### Encapsulation and Characteristics of pDNA-Encapsulated PLGA NPs

**FIGURE 2:**
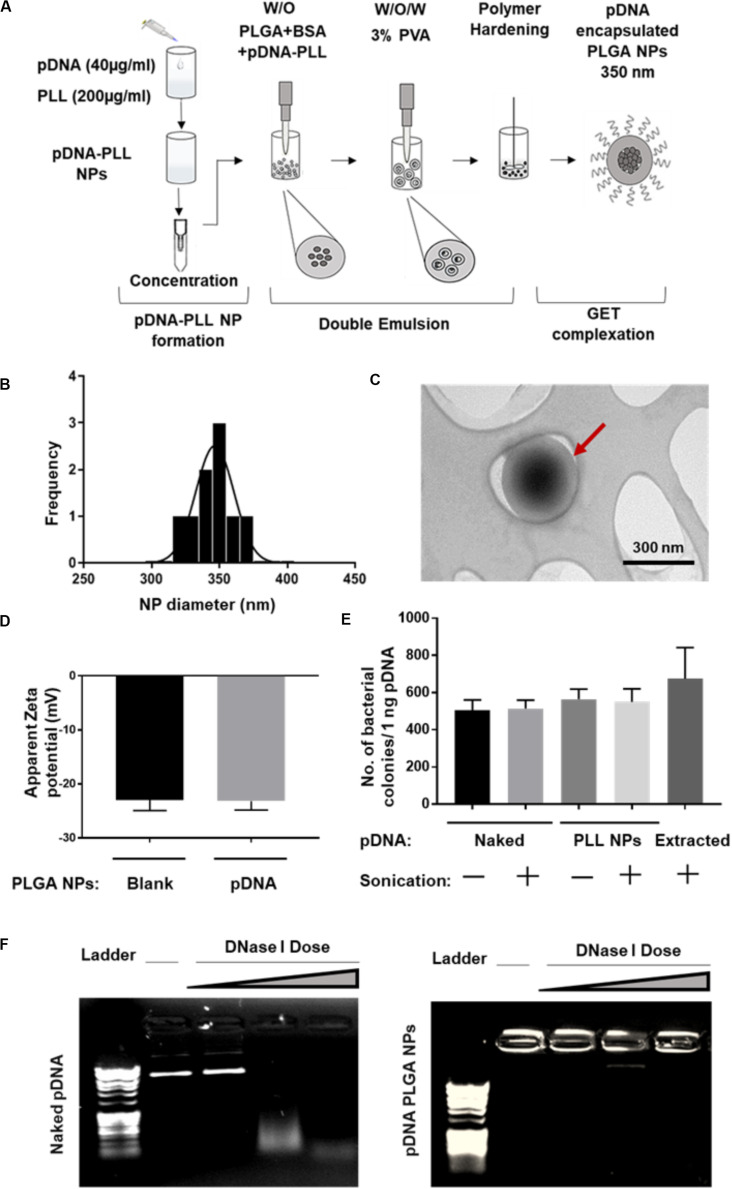
Characterization of pDNA-encapsulated PLGA NPs. **(A)** A simplified illustration of the process of pDNA encapsulated PLGA NP preparation and GET peptide complexation. **(B)** Representative PLGA NP size for both blank and pDNA encapsulated, shows homogenous population of the NPs measured by Zetasizer. **(C)** TEM micrograph of these NPs which confirms the size measured by Zetasizer and demonstrates the spherical, smooth surface of the NPs. Red arrow highlights individual NP. **(D)** The negative surface charge of pDNA-encapsulated PLGA NPs is comparable to blank NPs and excludes the possibility of PLGA NPs surface contamination with positively charged pDNA-PLL NPs. **(E,F)** Demonstrates the integrity of the encapsulated pDNA during the preparation process or the enzymatic treatment with DNase I respectively. Doses of DNase I tested were: 0.025, 0.0025, and 0.00025 U/μl with 15 min incubation time at 37°C.

Both pGluc- and pBMP2-PLL NPs were encapsulated in PLGA NPs using a double emulsion method (Figure 2A). This process yielded a minimum encapsulation efficiency (EE) percentage of ∼30% of the initial pDNA used. Blank PLGA NPs control were prepared by substituting the pDNA-PLL with water only. Blank PLGA NPs and pDNA-PLL NPs passed through the same double emulsion method but without the PLGA, were used as controls for measuring EE% and within transfection assays. The blank and encapsulated PLGA NPs are in the range of 350 nm in diameter with average PDI of 0.17 ± 0.02 (mean ± SD) shown by Zetasizer and TEM analyses (Figures 2B,C, respectively) with a negative surface charge (Figure 2D).

It was imperative to evaluate the possibility of unfavorable surface attachment of positively charged pDNA-PLL NPs on the negatively charged PLGA NPs by electrostatic interaction during the process of double emulsion. This effect might result in surface bound pDNA on PLGA NPs and consequently transfection as a result of binding rather than encapsulation of pDNA. To determine this, we considered and assessed many checkpoints during the formulation methodology. First, we compared the surface charge of pDNA-encapsulated PLGA NPs with blank NPs (Figure 2D); the charge of both NPs is negative which indicates no or little attachment of positively charged pDNA-PLL on the surface of PLGA NPs. Secondly, to control for the sedimentation of pDNA-PLL NPs during centrifugation and contribution to transfection, pDNA-PLL NPs were passed through the same process without PLGA (i.e., double emulsion, centrifugation and washing steps). No pellet of pDNA-PLL was found by centrifugation and we used these samples as controls in both measurements of the EE% and transfection of cells. As a result, no pDNA was detected in the supernatant. Additionally, even when complexed with GET peptides this did not result in significant detectable transfection in NIH3T3 cells. These controls confirm that pDNA-PLL NPs do not attach significantly to the surface of PLGA NPs and therefore hypothesized that almost all the pDNA is encapsulated within the PLGA NPs. We hypothesize that if pDNA is not encapsulated it does not sediment by centrifugation and is lost during the washing steps in the double emulsion process.

We next assessed the exposure of pDNA to the external microenvironment as a function of its encapsulation efficiency (Figures 2E,F). We confirmed that the encapsulated pDNA in PLGA NPs is prevented from migration through the gel and the PLGA protects the encapsulated pDNA from enzymatic treatment with DNase I, even at the highest doses which would be beyond anything physiological (Figure 2F). Moreover, there is a minimal effect of sonication on the integrity of pDNA at the rates used to prepare these NPs; this was confirmed by pDNA extraction and bacterial transformation assays demonstrating intact pDNA against sonicated and non-sonicated pDNA and pDNA-PLL NPs controls (Figure 2E).

### PLGA-GET NP Characteristics for Enhanced Intracellular Delivery

**FIGURE 3:**
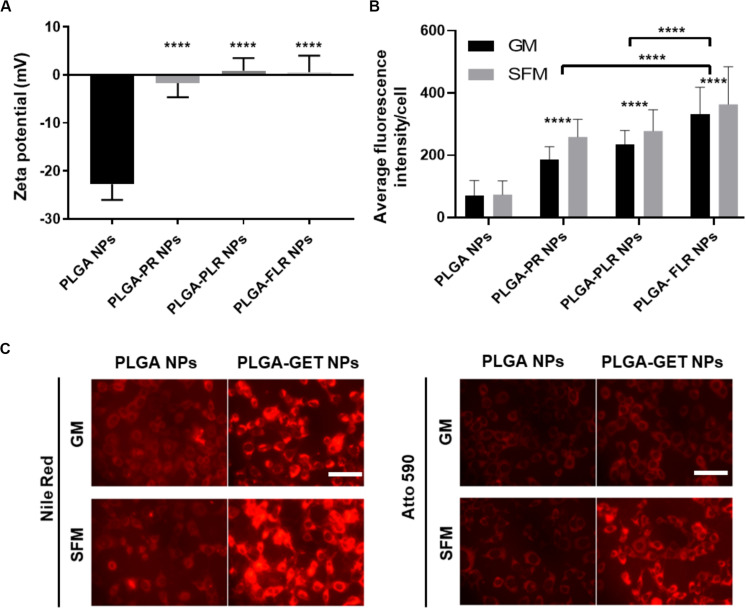
Enhanced delivery of PLGA NPs with GET peptides. **(A)** Interaction between GET peptides and PLGA NPs (Blank and pDNA-encapsulated) and confirmation of PLGA-GET complex formation through a shift in the surface charge of PLGA NPs measured by Zetasizer. **(B)** All variants of GET peptides enhance the delivery of PLGA NPs significantly in comparison to non-complexed PLGA NPs. PLGA-FLR NPs are associated with the highest levels of uptake followed by PLGA-PLR NPs quantified by flow cytometry analysis. **(C)** Fluorescence microscopy images of NIH3T3 cells after addition of Nile Red-, Atto590-encapsulated PLGA NPs with and without GET peptide complexation in 10% FCS containing media (Growth media, GM) or SFM. The peptide significantly enhances the PLGA NPs, especially in SFM. Scale bar 100 μm. The asterisk over the bars indicate significance to control (PLGA NPs), the asterisk over the lines indicate significance between groups. One-way and two-way Anova statistical analysis was used to generate the graph followed by Tukey test to determine significant differences between each mean. The data represented as mean ± SD. *P*-value were < 0.0001.

Upon complexation of negatively charged blank or pDNA-encapsulated PLGA NPs with positively charged GET peptides, the surface charge significantly changes from negative to a neutral or slightly positive charge (Figure 3A). We tested several variants of GET peptides namely P218R (PR), P21LK158R (PLR), and FGF2BLK158R (FLR); all three variants of GET peptide enhance the delivery of PLGA NPs to cells. However, the most significant enhancement in delivery as assessed by flow cytometry was observed when NPs were coated with LK15-containing peptides (i.e., PLR and FLR) over those lacking it (i.e., PR), or those with the FGF2B HS binding motif (over P21), and in general delivery was higher in SFM than in GM (Figure 3B). This was also confirmed with fluorescence microscopy (Figure 3C). We hypothesize this may be due to stronger electrostatic interaction within PLGA-GET complexes in SFM. The zeta potential change of PLGA-GET complexes incubated with increasing concentration of serum could potentially explain this (Supplementary Figure S1). Upon incubation for 15 min, PLGA-GET NP surface charge becomes more negatively charged with serum reaching approximately −10 mv in 10% FCS containing GM media. This effect is likely due to the binding of negatively charged serum proteins such as albumin with positively charged GET peptides on the surface; or that serum induces minimal dissociation of GET peptides from the NPs exposing some of the negatively charged PLGA NP surface.

### Optimizing the GET Peptide NP Coating for Efficient Cell Transfection

**FIGURE 4:**
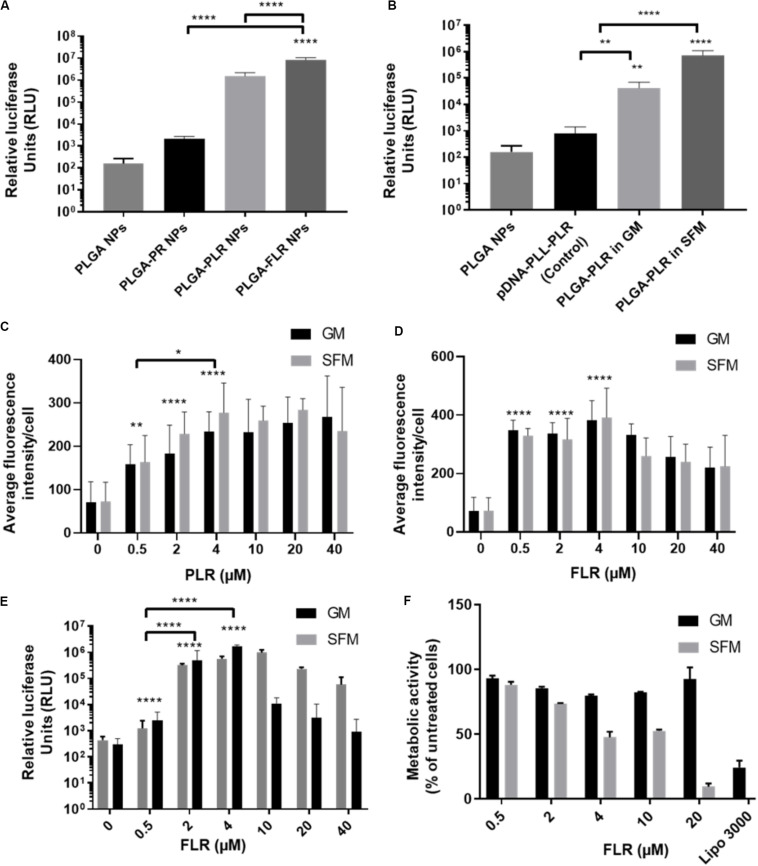
Enhanced transfection of PLGA NPs with GET. **(A)** Levels of transfection of different GET peptide complexation showing high levels of transfection of pDNA encapsulated PLGA NPs complexed with PLR and FLR in NIH3T3 cells, Corresponding 1 μg of pDNA content. **(B)** Validation of the encapsulated pDNA and the ability to transfect against controls (pDNA-PLL). Dose optimization of PLR **(C)** and FLR **(D)** peptides in IHMSCs. Transduction level increases with increasing the dose of both PLR and FLR. Enhancement reaches saturation at 4 μM FLR peptide. **(E)** Dose dependent enhanced transfection of IHMSCs, as a result of enhanced transduction by FLR. In line with transduction levels, with increasing FLR peptide concentration, the transfection levels improve until reaching saturation. **(F)** Cell metabolic activities 24-h after transfection with different doses FLR (highest transduction activity) in IHMSCs. In contrast to Lipofectamine 3000, FLR peptide show high margins of cytocompatibility especially in GM. The asterisk over the bars indicate significance to control (PLGA NPs), the asterisk over the lines indicate significance between groups. One-way and two-way Anova statistical analysis was used to generate the graph followed by Tukey test to determine significant differences between each mean. The data represented as mean ± SD. Where significance was ** or ****, *P*-value was <0.0021, or <0.0001 respectively.

pGluc-encapsulated PLGA NPs were used to assess transfection efficiency with GET peptide complexation. PR has been previously used to deliver many cargoes such as mRFP recombinant protein, magnetic NPs (Dixon et al., 2016; Markides et al., 2019), recombinant runt-related transcription factor RUNX2 (Thiagarajan et al., 2017) and MYOD for zonal myogenesis (Eltaher et al., 2016). However, when employed in the delivery of nucleic acids, PR is not sufficient to produce significant levels of transfection even with enhanced delivery characteristics. This is likely to be due to PR lacking LK15 which is the endosomal escape activity of the system (Saleh et al., 2010; Alkotaji et al., 2014; Ahmed, 2017) (Figure 4A). Corroborating experiments using fluorescent pDNA complexed with PR compared to PLR and transfected into NIH3T3 produced almost the same NPs in terms of physico-chemical characteristics and cellular internalization levels (by fluorescent microscopy and flow cytometry analysis) but only PLR showed effective transfection levels by luciferase assaying (Supplementary Figure S2). Additionally, it appears that fluorescent NPs are more diffusely localized in cytosol with PLR delivery and potentially accumulated more in the endosomes for PR (Supplementary Figure S2). Conclusively, the presence of LK15 is essential for pDNA-encapsulated PLGA NPs transfection. Therefore, GET peptides which bear LK15, i.e., PLR or FLR were utilized for transfection studies (Figure 4). When transfecting NIH3T3, complexation of PLR or FLR with PLGA NPs significantly increases the level of transfection by up to five orders of magnitude, in comparison to non-complexed PLGA NPs (Figure 4A). It is noteworthy that although there is some low degree of internalization of non-complexed PLGA NPs, this does not result in any levels of detectable transfection above background (10^2^ RLU). Moreover, SFM produced slightly higher transfection levels over GM conditions, which aligns with the enhanced transduction characteristics seen in the same media (Figure 4B). As we aimed to demonstrate the utility of our PLGA-GET system in biomedical applications, with a focus on osteogenic differentiation and regenerative medicine, we optimized the dose of both PLR and FLR peptide coating per weight of PLGA NPs for transfection studies of immortalized human Mesenchymal Stromal Cells (IHMSCs). The optimized dose was chosen based on levels of internalization (Figures 4C,D, for PLR and FLR respectively), and as FLR was superior this was taken forward for transfection (Figure 4E) and cell viability assays (Figure 4F). We found that with increasing FLR peptide, the transfection levels improved until reaching saturation at 4 μM (Figure 4E). There was slightly reduced cell viability in highly susceptible cell line such as IHMSCs at very high concentrations of FLR > 20 μM (for 0.2 mg PLGA NPs) especially in SFM (Figure 4F), which is also the case for other transfection systems (Jeong and Park, 2002).

Based on these assays, the optimized dose of 4 μM FLR (per 0.2 mg NPs) was employed for validating our systems use in differentiation studies. Interestingly, FLR-pDNA complexes have previously been shown as superior to PLR in transfecting hard to transduce cells (such as MSCs) when using smaller doses of pDNA (Osman et al., 2018). Importantly these studies confirm this also the case for PLGA NP formulations.

### Osteogenic Differentiation Assays

**FIGURE 5:**
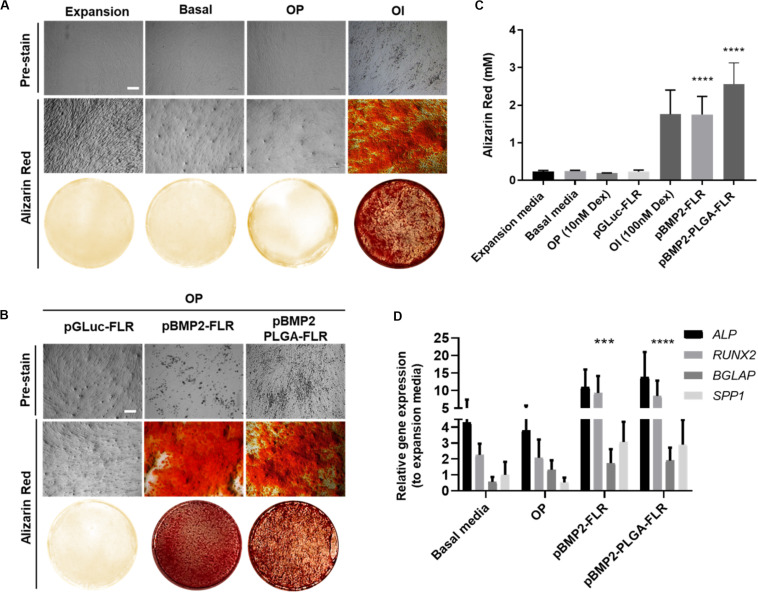
Osteogenic differentiation as a result of pBMP2 encapsulated PLGA- GET delivery. **(A)** Phase contrast microscopic images (pre-stain) and Alizarin Red stained microscopic and whole well images of control IHMSCs in expansion, basal, OP and OI media. **(B)** Phase contrast microscopic images (pre-stain) and Alizarin Red stained microscopic and whole well images produced dense calcium deposits in cultures transfected with pBMP2-FLR and pBMP2 encapsulated PLGA NPs in comparison to controls. Cells were cultured at 35k cells in 24-well plate format. Scale bar 100 μm. **(C)** Quantification of Alizarin Red stained calcium nodules; (mM) fold increase in dye absorbed calcium of transfected cultures in comparison to controls. **(D)** Significant activation of the osteogenic genes: *ALP* (Alkaline Phosphatase), *RUNX2* (Runt Related Transcription Factor 2), *BGLAP* (Bone Gamma-Carboxyglutamate Protein) also called *Osteocalcin*, and *SPP1* (Secreted Phosphoprotein 1) also called *Osteopontin* at week four from IHMSCs either treated with pBMP2-FLR NPs or pBMP2-encapsulated PLGA-FLR NPs in comparison to non-transfected cells. The graph was plotted based on expression fold change to cells cultured in expansion media only. One-way and two-way Anova statistical analysis were used to generate the graphs followed by Tukey test to determine significant differences between each mean. The data represented as mean ± SD. Where significance was *** or ****, *P*-value was, 0.0002 or <0.0001 respectively.

To determine efficacy of non-PLGA encapsulated pBMP2-FLR NPs in osteogenic differentiation (Figure 5A for assay), we confirmed that the previously published GET peptides alone can induce osteogenesis in IHMSCs with exogenous transgene-mediated BMP2 expression (Figure 5B middle section) (Supplementary Figure S3 for physico-chemical characterization) (Osman et al., 2018). Single transfection was used in osteopermissive (OP) media and a minimum dose of 0.5 μg of pBMP2-FLR transfection mix was required to induce osteogenesis. Next we compared the efficacy of pBMP2-PLGA-FLR and pBMP2-FLR NPs in this assay differentiation was confirmed for both systems. IHMSCs initially changed morphology from fibroblastic to polygonal shaped cells in the early stages of differentiation (week 1), followed by increased deposition of ECM and calcium nodules forming on cells observed by light microscopy starting from week 3 (Figure 5B pre-stain). This differentiation was also confirmed by Alizarin Red staining at week 4 (Figure 5B). Extracted Alizarin Red from stained wells was used as a relative quantification method of the calcium nodule numbers confirming that both published GET NPs (Raftery et al., 2019) and the PLGA-GET NPs can induce similar levels of oestogenesis (Figure 5C). Ten-fold increase in molar concentration of the absorbed dye by the calcium nodules was demonstrated for BMP2-transfected cells in comparison to controls in expansion, basal, OP media and those in OP media transfected with pGluc-FLR NPs.

Cultures in basal media, Osteopermissive (OP), or transfected with pGluc instead of pBMP2 resulted in no spontaneous osteogenic differentiation. Moreover, highly osteoinductive (OI) media with Dexamethasone produced similar levels of differentiation as those of pBMP2 transfected samples (Figure 5A). Furthermore, elevated levels of osteogenic specific genes in transfected cells with both pBMP2-PLGA-FLR and pBMP2-FLR were confirmed by Q RT-PCR. Importantly, late osteogenic marker, matrix synthesis and mineralization specific gene; *BGLAP* was significantly upregulated in the transfected cells over the control in basal media and OP (Figure 5D) (Lian et al., 1989; Ryoo et al., 1997; Thiagarajan et al., 2017). Cell viability assays showed minimal toxicity of pBMP2-encapsulated PLGA-FLR NPs during the course of transfection in the differentiation studies (Supplementary Figure S4).

## Discussion

Non-viral gene delivery vectors are known for their low efficiency when compared to viral vectors. Examples of non-viral systems include cationic polymers which act to both condense and enhance the delivery of nucleic acids. However, for enhanced gene delivery characteristics, high doses or highly positively charged variants are generally employed (Kadlecova et al., 2012; Hall et al., 2017). This high positive charge is directly related to cellular toxicity (Moghimi et al., 2005). Moreover, cationic agents only partially protect the nucleic acid cargo against enzymatic degradation (Roy et al., 2003; Hu et al., 2016). On the other hand, the use of effective versions of bio-inert hard polymers such as PLGA, would represent a significant step forward in non-viral gene delivery because of their advantages in attaining maximum DNase enzymatic protection and low toxicity (Han et al., 2000; Jin et al., 2014; Chen et al., 2016). PLGA is a gold standard in developing macromolecule-encapsulated particles such as for proteins and nucleic acids. Encapsulation protects the macromolecule from degradation by enzymes such proteases/nucleases and other environmental factors such as infected matrices and non-physiological pH (Pack et al., 2005; Ding and Zhu, 2018). Our demonstration here provides the foundation for developing future first-in-class pharmaceutical gene therapy product. However, such PLGA NPs poorly internalize and yield almost no transfection levels *in vitro* or *in vivo* when adequately removing and controlling for contaminating nucleic acids left from the fabrication process (Torchilin, 2008; Steinbach et al., 2016; Kim et al., 2019).

Modifications of CPPs such as GET peptides have improved nucleic acid delivery and are associated with low cytotoxicity when compared to competitor transfection agents such as Polyethylenimine (PEI) and Lipofectamine family agents (Life Technologies) (Grigsby and Leong, 2010; Kapoor et al., 2015). We have previously published our pDNA-GET NP system (Osman et al., 2018; Raftery et al., 2019), in which we demonstrated superior gene delivery, transfection efficiency and improved cell viability for lung and bone gene therapy applications. However, condensation of the plasmid with GET peptides to form a NP complex is not sufficient to fully protect the encapsulated pDNA from enzymatic degradation (Abbas et al., 2008; Osman et al., 2018), especially when applied *in vivo*. Therefore, in this work we encapsulated pDNA in the core of the PLGA NPs for almost total protection against DNases and the GET peptide system was complexed pDNA-encapsulated PLGA NPs by facile incubation to efficiently deliver high transfection levels into cell lines and human MSCs.

For encapsulation of nucleic acids, it is important that the process is controlled using proper experimental comparisons, which has been lacking in some previous studies using PLGA encapsulation (Capan et al., 1999; Tahara et al., 2008, 2010; Patil and Panyam, 2009; Zhao et al., 2013). There are several techniques to enhance the encapsulation of macromolecules such as nucleic acids in PLGA NPs (Mok and Park, 2008; Tahara et al., 2008; Danhier et al., 2012); one being the use of cationic reagents to condense and minimize the effective size of the macromolecule hence attaining higher encapsulation efficiencies. However, in previous studies the process is poorly controlled and these did not follow the dynamics of encapsulation and specifically address where the condensed nucleic acid were located; either being encapsulated in core or bound to the surface of the PLGA NP. It is highly likely that the small sized positively charged NPs (cationic polymer condensed nucleic acids) bind to the surface of negatively charged PLGA NPs through electrostatic interactions. The surface binding produces a slightly positive or neutral PLGA NPs as a result of charge neutralization; an effect identified by measuring the surface charge in comparison to blank NPs. In the present work, we have accounted for this possibility; moreover, we have applied other parameters to ensure that non-encapsulated DNA-PLL NPs do not sediment with the PLGA NPs during fabrication by centrifugation. To confirm that transfection was entirely attributable to encapsulated pDNA, we passed pDNA-PLL NPs through double emulsion process without the inclusion of PLGA. This control was used to quantify estimated pDNA-PLL NPs by DNA quantification and measure its effect on transfection. Both controls have successfully proven no surface attachment or sedimentation of DNA-PLL NPs occurred during the process of PLGA NP preparation and transfection is entirely due to encapsulated pDNA in the core of PLGA NPs. We believe our investigation is one of the first studies to take into account such controls for encapsulation.

Complexation of PLGA NPs with GET peptides significantly enhances the intracellular delivery and consequently transfection levels confirmed by reporter gene expression (pGluc). We employed a human MSC line that can differentiate to bone lineages given the correct stimulus (here ectopic BMP2 stimulation) to optimize the use of PLGA-GET NPs for bone regenerative medicine. It is known that BMP2 alone is not sufficient to induce human MSCs toward bone differentiation in *in vitro* conditions (Yang et al., 2003; Wang et al., 2016; Raftery et al., 2017; Loozen et al., 2018), and there are species differences between human and rodent MSCs in ease of specification (Diefenderfer et al., 2003; Osyczka et al., 2004; Acil et al., 2014). It is therefore important to culture hMSCs in a permissive but not inductive environment to test the activity of BMP2 gene delivery to trigger osteogenesis. We therefore added sub-threshold levels of Dexamethasone (10 nM) to generate osteopermissive (OP) conditions and media. It is important to note that native glucocorticoids are considered essential for initial morphological fibroblastic to polygonal changes for lineage specification (Jaiswal et al., 1997). We compared OP conditions to high Dexamethasone (100 nM) i.e., Osteoinductive (OI) media, which is capable of inducing differentiation without BMP2 supplementation.

Confirmation of osteogenic differentiation by Alizarin Red staining, upregulation of osteogenic genes and cell viability studies suggest that pBMP2 encapsulated PLGA-GET is an effective gene delivery system in osteogenesis with no significant cytotoxic effects, and comparative to our published studies (Osman et al., 2018; Raftery et al., 2019). We confirm that transfection activity is not due to excessive positive charge as it is associated with other non–viral gene delivery vectors. Indeed these PLGA-GET complexes are neutrally charged and the enhanced transfection is entirely mediated by the delivery characteristics of GET peptide, including the requirement for effective endosomal escape activity. This system has many other advantages compared to the current non-viral gene delivery vectors, including GET peptide delivery alone, as it combines the benefits of gene protection, efficient transfection, and the potential for prolonged storage and stability. These attributes are significant when developing and translating a pharmaceutical gene delivery product. Moreover, our system could be used to encapsulate and deliver multiple regulatory factors to harness synergistic effects of several genes in more complex 3D models of bone formation and for other tissues and disease focuses. Our study did not aim to show difference in levels of osteogenesis between pBMP2-GET and pBMP2-PLGA-GET transfection systems *in vitro*. Both NPs are highly effective at transfecting hMSCs and initiating osteogenesis, and now require extensive *in vivo* comparison, as in our previous studies (Raftery et al., 2019).

We successfully encapsulated pDNA in PLGA NPs to take advantage of the characteristics of PLGA; aiming to promote pDNA protection and GET peptide complexation for an off-the-shelf gene therapy product as a first-in-class technology. Importantly using hard PLGA NPs for gene delivery has the potential to be used *in vivo* to enhance local bone formation, with or without biomaterial scaffold constructs. Examples would include injection directly into defects or into soft tissue surrounding it to induce osteogenesis. Other applications, such as *ex vivo* transfection of MSCs in compromised inflammatory bone defects and cell therapy, or directly in bone infections that would require enhanced nuclease protection, could be exploited using this system. Clearly our demonstrations of this system must move *in vivo*; either demonstrating efficiency in regenerating critical-size defects, or in subcutaneous ectopic bone formation assays, which is now the focus of our work.

## Conclusion

We have shown that employing FDA-approved material PLGA NPs for gene delivery could be transformative by combining the properties of nucleic acid protection, low toxicity and translational advantages. When combining these with GET peptide-mediated delivery the levels of transduction and transfection are robust and potentially allow their use in many biomedical areas. A versatile system that can be used to deliver genes or genetic systems for specific diseases or trauma treatment could have significant impact; especially when applying gene augmentation and editing strategies to enhance future regenerative medicine and cellular therapies.

## Data Availability Statement

The raw data supporting the conclusions of this article will be made available by the authors, without undue reservation.

## Author Contributions

JD conceived and initiated the project, and also supervised the study. JD and AJ designed the experiments. AJ and JD conducted the experiments and wrote the manuscript. Both authors contributed to the article and approved the submitted version.

## Conflict of Interest

The authors declare that the research was conducted in the absence of any commercial or financial relationships that could be construed as a potential conflict of interest.

## Supplementary Material

FIGURE S1 Effect of serum albumin on PLGA-GET peptide interaction. Change in zeta potential of PLGA-GET NPs upon their incubation in increasing concentrations of FCS in SFM. The significant change in surface charge from neutral to negative Indicates serum albumin electrostatic binding to GET on or distant from the surface of PLGA NPs. The reduction in surface is charge is proportional to the amount of serum in the media.

FIGURE S2 Comparison of the physicochemical characteristics, intracellular localization and transfection of pDNA-PR and pDNA-PLR NPs. **(A)** The effect of lack of LK15 peptide in pDNA-PR NPs on their levels of transfection in comparison to pDNA-PLR NPs. pDNA fluorescent labeling does not affect the luciferase signal detected by luminometer; demonstrated by transfection in unlabeled pDNA-PLR NPs positive control published in Dixon et al. (2016), Corresponding 1 μg of pDNA. **(B)** pDNA condensation with PR and PLR produced NPs of the same size range measured by Zetasizer excluding the effect of variance in NP size on cellular internalization and transfection. **(C)** Both NPs show intracellular localization detected by fluorescent microscopy imaging after overnight transfection of NIH3T3 cells in GM. The pDNA-PR NPs show discrete endosomal fluorescent NP accumulation in contrast to more diffuse pDNA-PLR NPs in the cellular compartments. Scale bar 100 μm **(D)** Comparable intracellular levels of NPs quantified by flow cytometry analysis shows no significant difference between pDNA-PR and pDNA-PLR NP transduction levels.

FIGURE S3 Characterization of pBMP2-FLR NPs. Characterization of the physicochemical properties of pBMP2-FLR NPs indicates desirable **(A)** NP size range and **(B)** surface charge for intracellular delivery. **(C)** Confirmation of NP production and morphology by TEM imaging. Red arrows highlight individual NPs.

FIGURE S4 Metabolic cell activity during osteogenic differentiation. Metabolic activity of the transfected IHMSCs during the first 3 days of differentiation. The cells retain at least 70% of viability during highest transfection levels (post-transfection days 2 and 3).
