# Supplementary figures and images for: Efficient Delivery of Transducing Polymer Nanoparticles for Gene-Mediated Induction of Osteogenesis for Bone Regeneration
Source: Front Bioeng Biotechnol. 2020 Aug 5;8:849. doi: 10.3389/fbioe.2020.00849 (PMC7419434; doi:10.3389/fbioe.2020.00849)

Figure S1

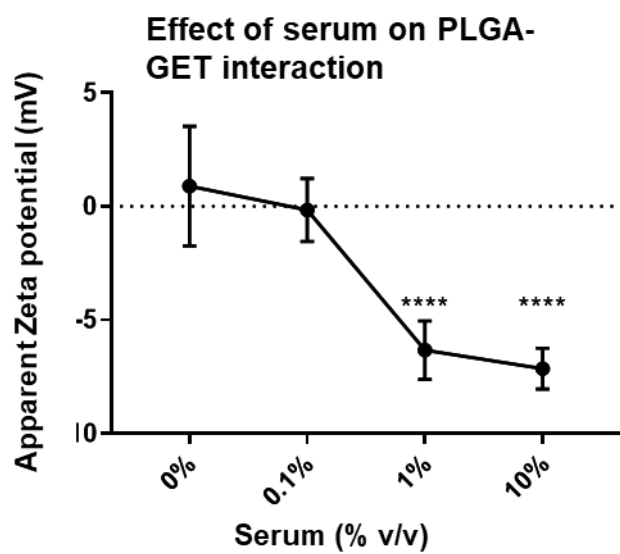

Figure S2

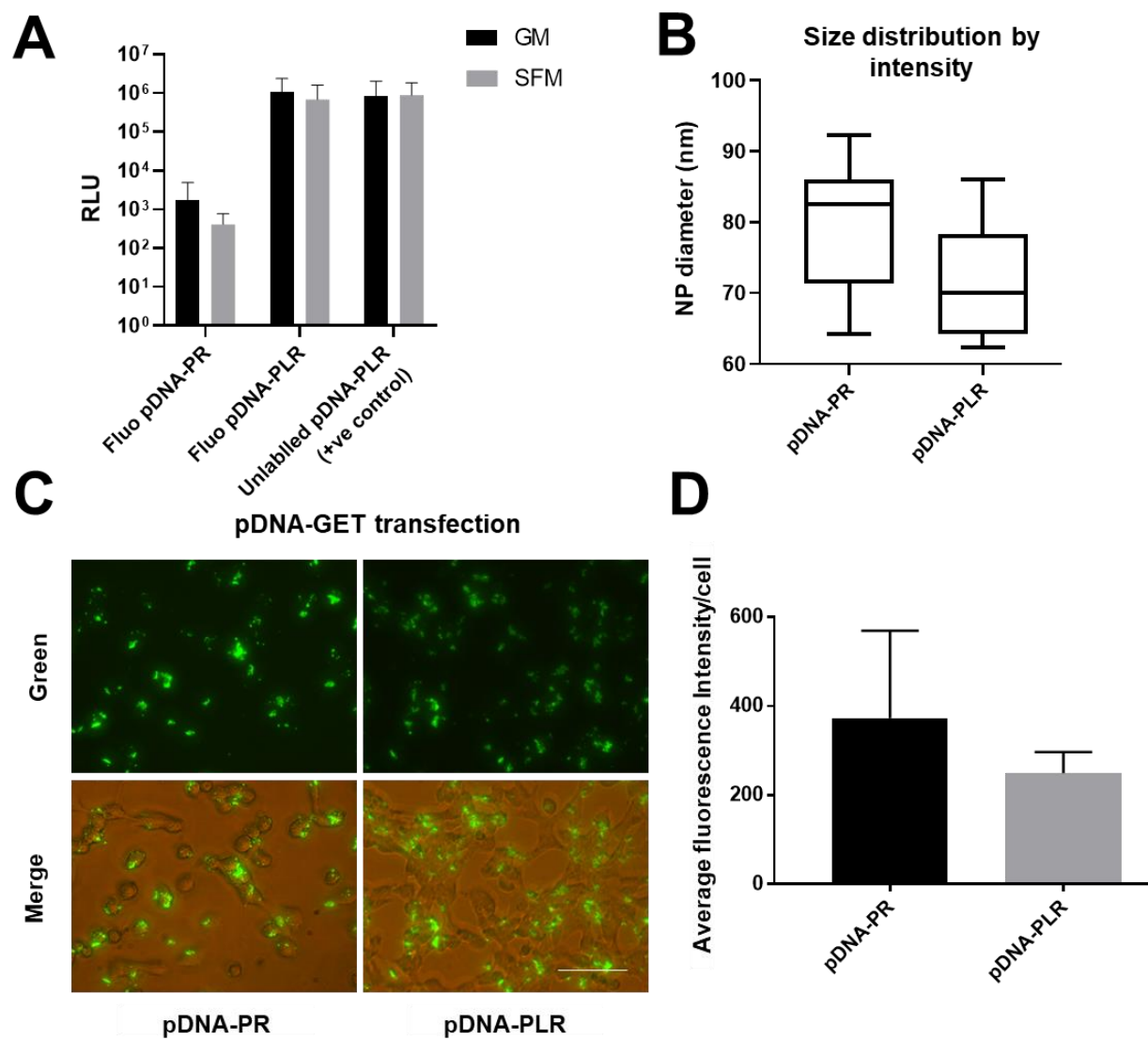

Figure S3

**A** pBMP2-FLR NPs size distribution by number

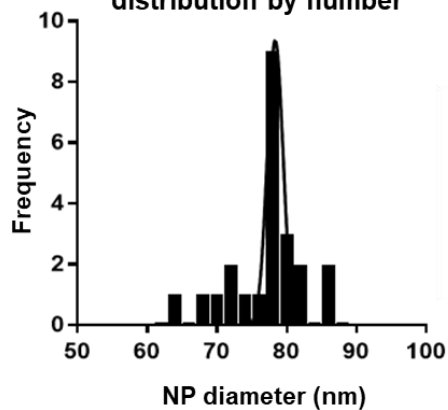

**B**

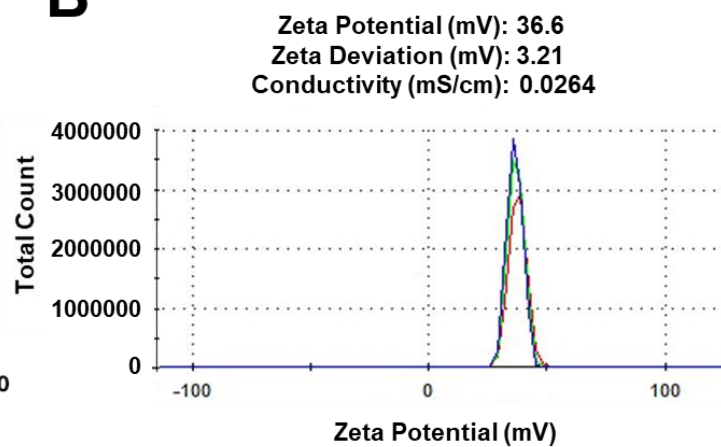

**C**

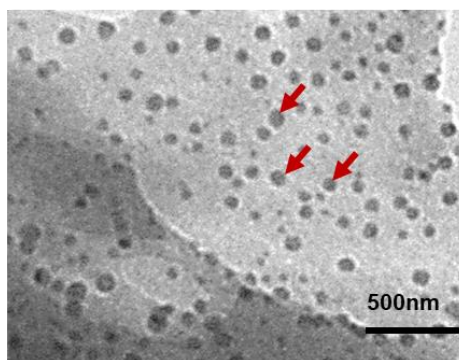

Figure S4

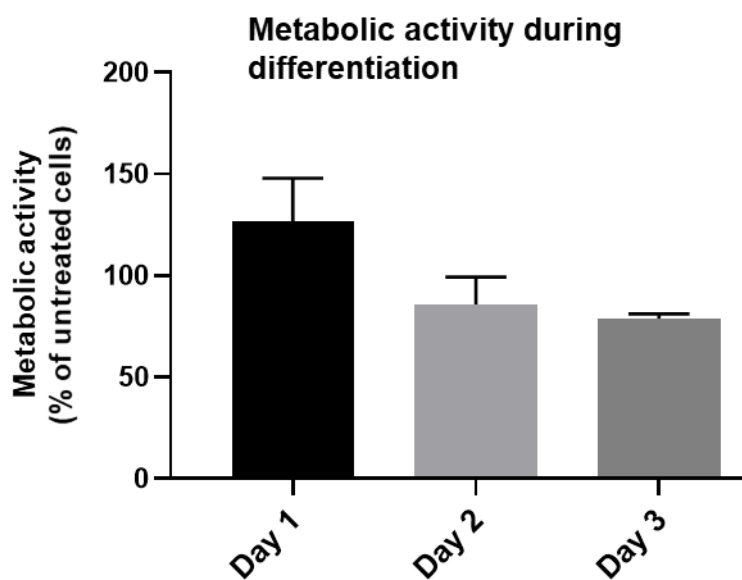

Supplement: FIGURE S1 — Effect of serum albumin on PLGA-GET peptide interaction. Change in zeta potential of PLGA-GET NPs upon their incubation in increasing concentrations of FCS in SFM. The significant change in surface charge from neutral to negative Indicates serum albumin electrostatic binding to GET on or distant from the surface of PLGA NPs. The reduction in surface is charge is proportional to the amount of serum in the media. [file Data_Sheet_1.PDF]
